# Perceived Cost Advantages and Disadvantages of Purchasing HIV Self-Testing Kits among Urban Tanzanian Men: An Inductive Content Analysis

**DOI:** 10.4172/2155-6113.1000725

**Published:** 2017-08-31

**Authors:** Larissa Jennings, Donaldson F Conserve, Jamison Merrill, Lusajo Kajula, Juliet Iwelunmor, Sebastian Linnemayr, Suzanne Maman

**Affiliations:** 1 Johns Hopkins Bloomberg School of Public Health, Department of International Health, Social and Behavioral Interventions Program, Baltimore, USA; 2 University of South Carolina, Arnold School of Public Health, Department of Health Promotion, Education, and Behavior, Columbia, USA; 3 Muhimbili University of Health and Allied Sciences, Dar es Salaam, Tanzania; 4 University of Illinois Urbana Champaign, Department of Kinesiology and Community Health, Champaign, IL, USA; 5 RAND Corporation, Santa Monica, CA, USA; 6 University of North Carolina at Chapel Hill, Gillings School of Global Public Health, Chapel Hill, NC, USA

**Keywords:** HIV self-testing, Financial benefits, Costs, Tanzania, Men, Qualitative

## Abstract

Impoverished men have lower rates of facility-based HIV counseling and testing and higher unknown HIV-positive status than women. Economic theory suggests that individuals will obtain an HIV test if anticipated benefits are greater than anticipated costs. Yet, few studies have investigated the range of financial preferences of HIV self-testing (HIVST) among poor men who decline testing or do not test regularly. Twenty-three interviews were conducted to qualitatively assess perceived costs saved and costs incurred from use of HIVST kits in infrequently- or never-tested Tanzanian men. All men were shown an HIVST kit and video. They were then asked about the costs associated with provider-led HIV testing, financial benefits and concerns of HIVST and willingness to pay for HIVST. Data were transcribed, coded and analyzed using inductive content analyses. We then grouped codes into perceived cost advantages and disadvantages and tabulated the range of prices men were willing to pay for a self-test kit. Perceived cost advantages of HIVST were avoidance of spending money to test in facilities, omission of follow-up fees, affordability relative to private clinics, and increased time for earning income and other activities. Men also discussed the imbalance of the financial benefit of accessing free, public HIV testing with the resources spent for transport, purchasing meals away from home and long wait lines. Perceived cost disadvantages of HIVST were prohibitive kit costs, required prior savings to purchase kits, expenditures relating to death and preferences for free provider-performed testing. Men were also concerned about the psychological costs of inaccurate results. HIVST willingness to pay varied among men. Men’s decisions to self-test for HIV takes into account expected financial gains and losses. Demand generation for HIVST among men should consider use of low fees or free HIVST, while emphasizing potential savings from reduced travel, clinical costs, or time way from work. Efforts are also needed to address anticipated emotional costs of HIVST, such as anxiety from kit errors, purchasing “death” or testing alone, which for some men was a substantial barrier.

## Introduction

National patterns on the uptake of HIV counseling and testing services (HCTS) in sub-Saharan Africa indicate that men have lower HIV testing rates than women [1]. The difference in HIV testing uptake between men and women is influenced by many factors, including the fact that many women test and initiate care during pregnancy in the context of antenatal services [2–4]. In general, men have less interaction with the health care system [5] and thus there are fewer opportunities to engage them in HCTS. As a consequence, a high proportion of HIV-positive men are unaware of their HIV status, and may engage in sexual risk behaviors that lead to HIV transmission [1]. The low rate of HCTS uptake among men compared to women also results in earlier mortality among HIV-positive men due to late-stage diagnosis, initiating antiretroviral therapy (ART) with lower CD4 cell counts, and having more advanced disease [6–9]. Unknown HIV status as a result of low uptake of HCTS additionally impedes use of prevention technologies for men, such as medical male circumcision, rectal microbicides, pre-exposure prophylaxis, and early ART initiation [10]. Learning that one is infected with HIV has been shown to lead to reductions in unprotected sex and sex with multiple partners, behaviors that contribute to the spread of the virus [11–17]. Therefore, to achieve the UNAIDS goal of 90% of all people living with HIV knowing their HIV status [18,19] and ultimately reducing HIV transmission, efforts are needed to identify innovative approaches to increase uptake of HIV testing among men, particularly in settings with high resource constraints and HIV prevalence.

HIV self-testing (HIVST) is a new approach that may offer a preferred and effective strategy to increasing testing rates in men [20–23]. HIVST is defined as any mode of HIV testing that allows a person to collect his own specimen in private, conduct a rapid antibody test, and be the first person to learn of the results [10,24]. HIVST differs from mobile- and home-based HCTS in that it is not conducted by a trained health care provider [25,26]. The oral fluid HIV self-testing kit has increasingly been piloted in sub-Saharan Africa and found to be feasible and acceptable in countries such as South Africa [25,27], Malawi [23,28,29], Kenya [30,31] and Uganda [32]. Several African ministries of health have also removed legislative bans on the sale of HIVST kits [27,33] and developed policy guidelines for HIVST in the general public [30,34]. The World Health Organization also released guidelines in 2016 to support the implementation and scale-up of HIVST [35]. Proposed advantages of HIVST are that it has the potential to reach untested individuals, including those who infrequently engage with the health care system – and thereby drastically reduce the number of undiagnosed HIV cases [8,10,26,36,37]. Proponents of HIVST also note that it is convenient, addresses stigma, privacy and confidentiality barriers to facility-based testing, can be provided at scale and at relatively low cost, and empowers individuals to make positive health decisions [10,25,26,36].

Specifically for men, HIVST could result in increased initial and repeat testing among those who have previously declined facility-based counseling and testing. It may also encourage joint testing among men with their sexual partner(s) [37–39], including men who have sex with men [MSM] [40,41]. Because in many traditional societies, men are also often breadwinners within households, an additional appeal of HIVST may be that it can reduce household testing costs relating to transportation to test sites or time and money lost waiting in lines [25]. In fact, low socio-economic status has been shown to contribute to low testing rates among men [42]. In addition, self-testing may be less costly than provider-based testing for the majority individuals who will test negative as well as for budget-constrained health systems [43]. In contrast, concerns regarding HIVST include incorrect use, misinterpretation of test results, possible harm to oneself or others after observing a positive result, coercion risks, and potential omission of care-seeking to confirm results [21,44].

Economic theory suggests that rational individuals will obtain an HIV test if the anticipated benefits are greater than the anticipated costs [45]. This would mean that an individual’s decision to test for HIV reflects a valuation of testing that exceeds both the expected financial costs of testing, such as clinic or laboratory fees, as well as the expected non-financial costs of testing, such as the physical discomfort from the test procedure, the psychological expense (i.e., stress, fear, stigma, guilt) of having a positive diagnosis, or the opportunity costs of lost fare, time, or productivity [21,46]. Individuals, including men, with low monetary resources may also be more sensitive to the cumulative costs, relative to the expected benefit, of initial and repeat testing [10,47,48]. However, few studies have investigated consumer views on the financial advantages and disadvantages of self-performing HIV tests, beyond the price of the test itself. The available economic studies relating to HIVST have primarily focused on willingness to pay for HIVST [21], economic characteristics associated with HIVST, such as employment and income [49–51], and cost-effectiveness of HIVST from the perspective of health systems [43,52,53]. However, less is known regarding perceived financial trade-offs of HIVST among economically-disadvantaged individuals, particularly sub-Saharan African men, who have high risk of HIV and are less likely to test using current models [10,54].

Therefore, the objective of this study was to qualitatively assess perceived cost advantages and disadvantages of using HIVST kits among infrequent and never HIV-tested urban men in Tanzania. Specifically, we examined men’s expectations about costs saved and costs incurred in using a self-test kit, including what men were able and willing to pay for HIVST. In this paper, we present findings from our qualitative assessment and discuss implications for increasing male uptake of HIV testing in resource-poor settings.

## Methods

### Study design

This study employed a cross-sectional qualitative research design using in-depth interviews with men living in Dar es Salaam, Tanzania. Data from the interviews were collected in 2015 as part of a follow-on phase to a prior quantitative survey conducted for a larger HIV prevention study [55]. The specific details of the quantitative survey are published elsewhere [55,56]. In sum, the survey examined the efficacy of a microfinance and peer health leadership intervention for HIV and intimate partner violence prevention among Tanzania men. This study consisted of the sample enrolled for the qualitative phase which comprised of sub-groups of men in Dar es Salaam who were purposively selected based on prior survey reports to include men with diverse characteristics relating to prior HIV testing and interest in using an HIV self-test in the future.

### HIVST and economic setting

Dar es Salaam is the commercial capital and largest city in Tanzania with an HIV prevalence of 6.9% among all city residents and 5.3% prevalence among urban men [57], compared to a national prevalence of 4.7% [1]. While Tanzania has made great strides in increasing HIV testing rates from 27% in men and 37% in women in 2008 to 47% in men and 62% in women in 2012 with the provision of free HCT through the Tanzania AIDS Prevention Program (TAPP) [58,59], uptake of HCT remains low and novel testing interventions are needed. Half of Tanzanian people ages 15–49 have never tested for HIV, and fewer than one-third of previous testers have been tested in the past 12 months [57]. In Dar es Salaam in particular, about one-third of men have been tested and received the results of their last test [1,60]. While the government of Tanzania has adopted a supportive policy of HIVST [61], self-test kits are not yet available for sale directly to consumers. In high-income countries, such as the U.S., the kit is sold over-the-counter for $40 USD. However, in African research settings, the most commonly available oral fluid-based self-test kits cost $3 to $12 USD [54,63–65]. The development of HIVST policies in Tanzania will have to account for high unemployment and concentrated areas of poverty, particularly in cities such as Dar es Salaam. An estimated 22% of city residents are unemployed, and many (63%) of the city’s employed residents rely on low-wage, self-employment. Urban men rely on work in wholesale or retail, such as repairing motor vehicles, as well as construction and transportation [62].

### Participant recruitment

Eligible participants included men aged 15 years and older, who socialized in fixed locations locally referred to as “camps,” and were willing to provide contact information during the survey phase of the study for future follow-up assessment. Camps are social gathering places where networks of mostly men frequent and have about 30–35 members and an average lifespan of 8 years [66]. They typically have elected leadership and some require membership fees to belong. We used camps to recruit urban men for the prior quantitative survey of which a sub-set were purposively selected for the qualitative phase. Camps were identified, mapped, and characterized using the Priorities for Local AIDS Control Efforts (PLACE) method, a venue-based sampling methodology that was developed as a surveillance tool for high-transmission venues [55,67,68]. A sub-set of men who completed the survey and reported being sexual active and having ever or never tested for HIV were contacted and informed about the qualitative phase of the study over the phone before scheduling the in-depth interviews.

### Data collection

All interviews were conducted in Kiswahili by two interviewers who were skilled in conducting qualitative research. Both interviewers received training on the objectives of the qualitative inquiry. The trained interviewers also watched a HIVST video and were each given a Calypte AWARE TM HIV-1/2 rapid oral fluid self-test to ensure that they were familiar with HIVST. This self-test is an accurate and easy-to-use rapid test for the detection of antibodies to HIV Type 1 and Type 2 in human oral fluid specimens [69]. The AWARE test has been evaluated with over 3,400 subjects and has an overall sensitivity of 99% and specificity of 100% [70,71]. The procedure for using the self-test consists of using an oral swab to collect oral mucosa in the mouth by rubbing the swab above the teeth against the outer gum and placing it into a sample buffer for mixing. The swab is then removed and discarded, followed by removing the test strip from the foil pouch and placing the test trip in the sample buffer mixture and reading the test result between 20 to 45 min later.

An open-ended interview guide was used to query participants on their perceptions regarding the cost advantages and disadvantages to HIV self-testing. To ensure that participants were knowledgeable about HIVST and referring to the correct kit, they were shown an HIVST kit and video before starting the HIVST section of the interview. The three-minute video demonstrated a young man opens the HIVST kit, reads the instructions, performs the self-test, and properly disposes of the kit. The video also provided guidance on how to interpret different test results and the importance of seeking confirmatory tests at a clinic. We then asked each man about the perceived costs associated with HIV testing in general, whether he was familiar with oral HIV self-testing, the perceived financial benefits and concerns of HIVST including any costs saved or costs incurred, and how much the participant would be willing to pay for HIVST kits. Each participant contributed to a maximum of one in-depth interview and was provided 10,000 Tanzanian shillings (TSH), the approximate equivalent of $4.50 U.S. dollars (USD) for his time. The interviews lasted for 30 to 60 min and were recorded and transcribed in Kiswahili, then translated to English.

### Data analysis

We used an inductive content analysis methodology, a technique used in qualitative research to categorize verbal data based on themes that emerge from the raw data (i.e., inductive reasoning), rather than by previously structured or specified hypotheses (i.e., deductive reasoning) [72,73]. Inductive content analysis is appropriate for research with little to no prior studies relating to the research question [73]. We chose this approach given the limited number of studies on perceived cost advantages and disadvantages of HIVST in sub-Saharan African men. Our analysis was conducted in two phases: a descriptive phase and an interpretive phase. In the initial descriptive phase, we aimed to determine what was said by the participants. Based on a close reading of a subset of transcripts, a list of descriptive categories was developed and applied using Dedoose (www.dedoose.com) online software to label data segments. These categories included: cost of HCTS, perceptions of the self-test kit, cost of self-testing, cost trades, willingness to buy self-test, not willing to buy self-test, price range, benefits of self-tests and challenges/harms of self-test. We then extracted and grouped text sets by category. This process allowed us to reduce the verbal data into more manageable sections for identifying patterns across participants related to cost perceptions. This also enabled us to focus our interpretive coding on text segments that were relevant to the research questions.

In the second interpretive phase, we aimed to assess what was meant and implied by participants within each of the categories. During this phase, we manually applied coding to each of text sets in a Word document, by writing analytical notes in the margins of the text while reading it and developing a short phrase to represent the interpreted meaning of a specific statement [74]. New codes were created and revised as new meanings emerged. An interpretive code list was then generated and used to code all of the transcript segments. We purposively aimed to characterize dominant themes that were frequently described by participants and subtle themes described by fewer participants. There were a total of seventeen interpretive codes applied, which we grouped into perceived cost advantages and perceived cost disadvantages (Table 1). As a final step, we extracted all participant statements regarding the price he was willing, unwilling, able, or unable to pay for a self-test kit. We then tabulated the average minimum and maximum price participants were willing and able to pay.

### Ethical approval

The study’s procedures and instruments were approved by the University of North Carolina (UNC) at Chapel Hill Institutional Review Board (IRB) and the Muhimbili University of Health and Allied Sciences (MUHAS) Senate Research and Publications Committee.

## Results

### Participant characteristics

A total of 23 men were enrolled in the qualitative phase of the study (Table 2). The mean age was 27.3 years (± 6.5), ranging from 20 to 51 years old. About half (n=12, 52%) of participating men had attained primary school education; 39% (n=9) had obtained secondary education; and 4% (n=1) had higher than secondary school education. An equal amount of men were married or cohabiting (n=11, 48%), single (n=11, 48%) as compared to having a non-cohabiting primary sexual partner (n=1, 4%). Employment rates were moderate with 65% (n=15) of men reporting being self-employed compared to 9% (n=2) who were employed by another person. However, a quarter (26%, n=6) of men were unemployed. Approximately half (57%, n=13) had obtained an HIV test at least once in their life, and 48% (n=11) had been tested for HIV within the past 12 months. The mean number of HIV tests obtained in the past 12 months was 2.6 (± 2.9), ranging from once to 11 times. The mean number of sexual partners in the past 12 months was 1.8 (± 1.0), ranging from 1–4 partners. Most men (78%, n=18) had no prior knowledge of HIVST. However, of those who had (22%, n=5), 80% (n=4 out of 5) had self-administered an HIV test. Willingness to HIV self-test in the future was relatively high (65%, n=15).

### Summary of thematic findings

We identified five perceived cost advantages to using HIVST kits among participating urban men (Table 3), as well as five perceived cost disadvantages of HIVST were identified (Table 4). These are summarized below with example quotations noted in Tables 3 and 4, respectively.

### Perceived cost advantages

#### Affordability relative to private clinics

The most commonly stated cost advantage of using an HIVST kit was that it would be more affordable than seeking HCTS at private clinics or hospitals that charge fees for this service. This sentiment was expressed both if HIVST kits were available by purchase at low price and offered for free. However, the price advantage of HIVST kits did not apply in comparison to government hospitals or mobile clinics (referred to as “caravans”) for which men reported HCTS being offered free of charge.

#### Increased time for earning and other activities

The second most commonly mentioned advantage was that HIVST would enable men to continue managing their own time and engage in the activities that were most important to them, such as earning income. Slightly over half of men were self-employed, and therefore valued that HIVST would offer an option to test for HIV without losing time on the job or doing domestic activities. Clinic-based testing was described as a waste of time, even up to a full day, of travel or waiting in line (referred to as “queues”) by men who had tested for HIV previously as well as those with no HIV test history. One man, who worked as a driver, described how much his time was valued for earning income – to the extent that he often ate and toileted in his vehicle. In this regard, some men had limited options to leave work to test for HIV elsewhere. The HIVST kit (referred to as “instrument”) was viewed as a convenient and non-disruptive testing option.

#### Avoidance of spending money to test in facilities

A more subtle cost advantage proposed by men was that using an HIVST would reduce the total costs of accessing test services. The majority of men who described this advantage had never tested for HIV using current clinic-or community-based models. Reductions in test costs were expressed in two ways. Firstly, even if HIVST kits were purchased at local kiosks (or “shops”), there was an expectation that the purchase price would be less than the costs of obtaining bus fare, acquiring meals on the road, and/or fees for service at private sites. Secondly, using an HIVST was expected to result in fewer people attending clinics for HCTS which would result in shorter waiting times – reducing one’s non-productive time or the need to purchase meals away from home.

#### Omission of fees for follow-up visits

Another cost advantage sometimes expressed by men was that HIVST would eliminate the need (and possibly the time and money) for returning to a clinic to obtain initial or confirmatory results. As two men stated, following the disposal of the test kit, they would know of their serostatus without being obliged to expend any additional effort or anguish. However, in some cases, the opposite view was stated. For example, some men indicated that an HIVST result would still have to be verified by a facility-based health provider and any costs related to follow-up would still apply.

#### Imbalance of benefits of free facility services with costs to access them

Men commonly discussed the imbalance of the financial benefit of accessing free HCTS at government hospitals with the financial costs of paying for travel and losing time to access those free services. As two men mentioned, testing centers were free of charge, but one had to spend money to get to them. As such, provision of free HIVST kits provided a conceivable fee-neutral advantage that additionally eliminated travel expenses. However, one man expressed skepticism that this imbalance would be addressed by HIVST as they were largely unavailable in Tanzania. In addition, mobile clinics and supervised home-based testing were being implemented to reduce financial barriers due to distance.

### Perceived cost disadvantages

#### Preference for free provider-performed tests

The majority of men were aware of free HCTS administered by health professionals and indicated that, in general, there were no direct costs to HIV testing. The concept of cost of testing was often limited to the presence of fees or not. Therefore, fee-neutral provider-performed testing was often preferred over self-testing if the latter required high payments. In other cases, however, non-financial costs were mentioned, such as physical discomfort from the test itself or the inconvenience of return visits. However, these non-financial costs were not perceived to outweigh the burden of test fees. For men who lacked or were unable to acquire resources, it was considered better to save money and go to a free clinic.

#### Prohibitive and expensive kit costs

Another common cost disadvantage was the potentially high and prohibitive pricing of self-test kits, which was expressed primarily by men who had never tested for HIV. Disapproval of high-priced kits was often posed in regards to prices they were unwilling or unable to pay, as well as a belief that HIVST should be offered at an affordable price so that the poorest individuals could self-test. Some men also indicated that the benefits of self-testing, such as saved time or money, would be questioned and undermined if the costs of the kits were too high.

#### Required prior savings and value to purchase kits

A less commonly discussed disadvantage of HIVST kits was the necessity of having saved resources or other financial assistance to purchase them. Some men implied not being able to afford HIVST kits presently, but intending to be financially-able in the future. They indicated needing to “prepare” for “that money” or referred to budgeting savings or asking relatives for resources. In other cases, being financially ready to engage in HIVST may have included taking time away from home/work, acquiring transport or arranging an appointment to acquire the kit.

Men also stated that having sufficient information on the benefits of HIVST device would be necessary to fully appreciate its value, especially for men who had never tested for HIV. Being educated on the advantages of HIVST was viewed as more critical than addressing kit pricing alone, as each person would undergo his own accounting of the gains and losses from HIVST.

#### Concerns regarding the psychological costs of inaccurate test results

There were also concerns regarding the psychological costs (i.e., worry, doubt, skepticism, distrust) one would incur from obtaining inaccurate HIVST results. This concern was particularly present for men who were less-educated. Having a false-negative or false-positive result was seen as a higher risk when relying on HIVST. Some men were skeptical of the kit itself, while others were not confident in their own capacity to correctly administer it.

#### Consequences of expenditures relating to death or that were unwise

A final cost disadvantage was the concern that purchasing an HIVST kit was congruous to purchasing “death”. This sentiment was expressed both solemnly and jokingly as an unconditional reason to avoid HIVST. Some men remarked that few people could be convinced to buy an item related to death. Another consideration was that purchasing HIVST kits was an unwise expenditure since the test may be of poor quality, unreliable, or error-prone. Some men felt that others would prefer to see to a doctor than spend money on a device they may not know how to use.

#### Ranges in willingness to pay for self-testing kits

Twenty-two (n=22) of the 23 participating men stated the price they were willing to pay for HIVST kits (Table 2), ranging from 0 to $9.20 USD (0 to 20,000 TSH). The largest proportion of men (n=10; 45%) were willing to pay the highest range of $6.91 to $9.20 USD (15,000 to 20,000 TSH). Of the remaining willing-to-pay participants, 9% (n=2) were willing to pay $4.61 to $6.90 USD (10,000 to 14,999 TSH); 14% (n=3) were willing to pay $2.30 to $4.60 USD (5,000 to 9,999 TSH); and 18% (n=4) were willing to the lowest range, $0.01 to $2.30 USD (1 to 4,999 TSH). Three participants (14%) indicated that they were not willing to pay for HIVST kits ($0 USD, 0 TSH).

## Discussion

To our knowledge, this is the first study to qualitatively examine perceived cost advantages and disadvantages of using HIVST kits among sub-Saharan African, urban men. We found that when asked to consider the prospect of using a self-administered HIV testing method, some men considered HIVST, provided for free or for a small fee, as a potentially more economical option than venue-based testing given the reduction in transport costs and the opportunity costs of lost income associated with time spent at the clinic. Other men expected HIVST to be a potentially more costly option due to the price of the kit itself or as a result of non-financial costs relating to test inaccuracy and distress. Men also perceived that cost advantages were omission of fees for follow-up visits and affordability relative to private clinics. Perceived cost disadvantages of HIVST were prohibitive kit costs, required prior savings to purchase kits, consequences of expenditures related to death and preference for provider-performed tests which were provided freely in hospital and clinical settings.

These findings point to some important policy implications for increasing uptake of HIV testing in men in resource-poor urban settings. One implication not surprisingly relates to the sensitivity of several men to price of the kit itself – and the appeal of free or low-cost HIVST. Our findings suggest that there is a potential large market for low-cost and easy to use HIVST kit among Tanzanian men. Nearly half of men (45%) were willing to pay up to $9 USD for HIVST, and the majority (68%) was willing to pay $2 to $5 USD. This is within the price range thus far of willingness to pay for HIVST in other low-income country settings [18] and within the HIVST kit price range, $3 to $12 USD, that has been negotiated in research settings in sub-Saharan Africa [54,63–65]. Men in our study suggested that they had sufficient resources to cover reasonable kit costs, or were willing to save money over time to purchase HIVST kits in the future. However, other men expressed concern about potentially high fees and preferred to use existing free clinic-based HCTS than purchase HIVST kits. This was a substantive concern that would likely impact utilization as approximately one third (32%) of men were either not willing to pay for HIVST kits or willing to pay only $2 or less. The direct purchase price of the HIVST kit was more salient among men than the related but less tangible costs such as time spent at clinic or costs for meals away from home. From a consumer perspective, kit pricing is a reasonable concern. Previous research has shown that the unit cost of an HIVST kit accounts for half of the total costs of HIVST for consumers [28] and that kit price is a barrier to HIVST acceptability [51]. Despite the appeal of HIVST in reducing costs related to clinic travel and waiting time, addressing cost concerns of the price of HIVST by providing free or subsidized HIVST kits may be necessary to fully reduce testing barriers in low-income men.

A second implication relates to other HIVST cost perceptions, not related to the price of the kit itself. Research has shown that men who perceive costly trade-offs are less likely to utilize HIVST or other test strategies [42]. In our study, those costly trade-offs included other financial and opportunity costs relating to facility-based testing, such as waiting time, lost earnings or travel expenses, which would favor use of HIVST. On the other hand, the perceived trade-offs of non-financial and emotional costs relating self-testing, such as the distress of having unreliable results or making a death-related purchase disfavored use of HIVST. These findings suggest that men’s individual cost structure (i.e., the process for determining the expected gains and losses) for deciding whether to self-test considers multiple trade-offs [46,75]. Men with the highest opportunity cost (i.e., those who face significant resource constraints or those who face high potential lost earnings in time) may prefer free or low-cost testing. Such men may find greater utility in seeking HIVST as a result of convenience, privacy, and total expense. In fact, HIVST users have been found to incur fewer non-clinical costs or missed days from work [28]. On the other hand, the expected benefits of HIVST for some men did not outweigh the anticipated losses of HIVST. Absorbing opportunity costs to access otherwise free, clinic-based HCTS was preferred in lieu of paying for self-test kits or experiencing emotional costs such as anxiety due to fear of false results or discomfort in testing without professional support. HIVST may therefore be ideal for men who decline or do not frequent testing facilities and perceive cost disadvantages of HIVST to be low. Other test options beyond HIVST may be needed for men who decline testing in facilities and perceive high financial and non-financial costs from HIVST.

As a result, the findings from this study could guide how HIVST is implemented. For example, research has shown that HIV diagnoses made in non-clinical settings, such as mobile vans or peer networks, are less effective in linking newly-identified cases to HIV care and treatment [51]. In our study, a misperceived advantage of HIVST among a few men was the omitted need for follow-up clinical visits. Therefore, information in the packaging and instructions of HIVST kits on how to link to low-cost post-test counseling and confirmatory testing would be essential. Adding HIVST post-test counseling to existing PEPFAR-funded HIV hotlines in Tanzania may also prove beneficial. Our findings also suggest that different strategies may be needed to increase male uptake of HIV testing across economic strata. Information campaigns could make more salient the convenience provided by HIVST for men with high opportunity costs of traveling to a clinic for testing, including emphasis on the use of saved time and money towards other HIV prevention behaviors, such as condom use or repeat testing. For men who are most sensitive to the price of the kit itself, and less concerned about clinic travel or waiting time, subsidies to reduce or remove direct kit costs may be most effective. For men who find the expected emotional and social costs of HIVST too great, it will be important to have interventions to decrease test anxiety, low test self-efficacy, or fear of ominous results. There may be a need to further address non-cost-related barriers to HIV testing in facilities, for never or infrequent testers who still prefer this strategy. Ultimately, our research underscores that new test options like HIVST will need to account for the range of cost perceptions and cost trade-offs (such as paying for self-test kits in order to save time and travel expenses) in order to successfully increase uptake of HIV testing services.

## Limitations and Strengths

This study was limited by some factors. Our findings represent men’s hypothetical views on cost advantages and disadvantages of HIVST. As HIVST is not yet available to consumers in Tanzania, the majority of participants did not have lived experience negotiating the cost preferences discussed in the interviews. To counter this concern, we showed all men an HIVST kit and video to help them consider its worth and how they would use the self-test. Many men also had prior experience receiving HCTS in facilities and were thus able to extrapolate cost factors relating to decisions to self-test based on their previous clinical experiences. We were also limited by the cross-sectional nature of our qualitative inquiry and were unable to longitudinally assess how changing cost perceptions influence uptake of HIVST, or how prior HIVST use impacts subsequent accounting of cost gains or losses. Without a known price of HIVST kits, men may have also over-emphasized affordability concerns. Finally, the study was based on a small number of men which may have limited the transferability of our results is therefore limited by the small sample size and may not be applicable to men who are not camp members.

Nonetheless, social camps are common in Dar es Salaam and recruiting men from these venues enhanced the generalizability of our research. This study also included other strengths. Our use of an inductive analytical approach allowed themes to merge from the data that were based on dominant and subtle views expressed by men. The study also included a diverse and heterogeneous set of narratives from a population that is under-studied in the economics-related HIV literature. Beyond the price of HIVST that men were willing to pay, this study further informs our understanding of perceived costs incurred and cost saved – and the trade-offs of those factors in decisions to self-test among low-income men who decline or infrequent facility-based testing.

## Conclusion

Men’s decisions to self-test for HIV takes into account expected financial gains and losses. Implementing low fees or free HIVST may increase uptake of HIVST, including enhancing the perceived cost advantages of self-testing, such as reduced travel, clinical costs and time away from earning income. Efforts are also needed to address anticipated emotional costs of HIVST, such as anxiety from kit errors, purchasing “death” or testing alone, which for some men was a substantial barrier.

## Figures and Tables

**Table 1:** Categories, themes and codes emerging from men’s interviews about cost perceptions of HIV self-test kits.

Category	Rank	Emergent Themes	Codes	Frequency of applied code out of 121 applications [n (%)]
**Cost Advantages**	****	Affordability relative to private clinics	Kit saves money that would be used at facilities	14 (11.6%)
			Clinic-testing isn’t always free	6 (5.0%)
	***	Increased time for earning and other activities	Kit saves time relative to other test models	14 (11.6%)
	**	Reduction in money lost to test in facilities	Kit saves money that would be used at facilities	5 (4.1%)
	**	Omission of fees for follow-up visits	No need for return for results with self-test	4 (3.3%)
	*****	Imbalance of benefits of free facility services with costs to access them	Availability of free clinic-based HCTS	13 (10.7%)^a^
			Implicit cost trades	12 (9.9%)
			Knowing good health is priceless	1 (0.8%)
**Cost Disadvantages**	*****	Preference for free provider-performed tests	Availability of free clinic-based HCTS	13 (10.7%)^a^
			Hospital is where most are tested	4 (3.3%)
			Cheaper to go to clinic during financial hardship	2 (1.7%)
	****	Prohibitive and expensive kit costs	Unaffordable kit price	10 (8.3%)
			Providing access to HIVST to the extremely poor	8 (6.6%)
	****	Concerns regarding the psychological costs of positive or inaccurate results	Fear of positive, inaccurate test results	13 (10.7%)
			Fear of unintended disclosure of positive results	5 (4.1%)
	***	Required prior value and savings to purchase HIVST kits	Needing to save pre-purchase	6 (5.0%)
			Need to be informed of value in order to consider buying	9 (7.4%)
	**	Consequences of death-related and unwise expenditures	Buying self-test is like buying death	5 (4.1%)
			Distrust in test equipment, not worth buying	4 (3.3%)

[a] This code was used in the interpretation of two themes, but is only counted once in the number of applications

**Table 2:** Frequency of participant demographic, employment, HIV testing, and willingness to pay characteristics (N=23 interviews).

Demographic Characteristics	

**Mean age in years** (+**SD**)	27.3 (± 6.5)

**Minimum, maximum age in years of sample**	20, 51

**Gender**	
**Male**	23 (100%)
**Female**	0 (0%)
**Other**	-

**Education**	
**No formal education**	1 (4%)
**Primary**	12 (52%)
**Secondary**	9 (39%)
**Higher than secondary**	1 (4%)

**Marital status**	
**Single**	11 (48%)
**Married/Cohabiting**	11 (48%)
**Girlfriend/boyfriend**	1 (4%)

**Employment Characteristics**	

**Employment**	
**Employed by other(s)**	2 (9%)
**Self-employed**	15 (65%)
**Unemployed student**	1 (4%)
**Unemployed non-student**	5 (22%)

**Worked in the past 7 days**	
**Yes**	19 (83%)
**No**	4 (17%)

**HIV Testing and Willingness to Pay Characteristics**	

**HIV tested in lifetime**	
**Yes**	13 (57%)
**No**	10 (43%)

**HIV tested in the past in 12 months**	
**Yes**	10 (43%)
**No**	12 (52%)
**Missing**	1 (4%)

**Mean number of HIV tests obtained in past 12 months (± SD)**	2.6 (± 2.9)

**Minimum, maximum number of HIV tests obtained in past 12 months**	1, 11

**Mean number of sexual partners in past 12 months**	1.8 (+ 0.95)

**Minimum, maximum number of sexual partners in past 12 months**	1, 4

**Ever heard of oral HIV self-test**	
**Yes**	5 (22%)
**No**	18 (78%)

**HIV self-tested in lifetime**	
**Yes**	4 (17%)
**No**	19 (83%)

**Willingness to HIV self-test in the future**	
**Yes**	15 (65%)
**No**	8 (35%)

**Willingness to pay for HIV self-test in the future**^a^	
**Not willing to pay ($0 USD; 0 TSH)**	3 (14%)
**Less than $2.30 USD (<5,000 TSH)**	4 (18%)
**$2.30-$4.60 USD (5,000–9,999 TSH)**	3 (14%)
**$4.61-$6.90 USD (10,000–14,999 TSH)**	2 (9%)
**$6.91-$9.20 USD (15,000–20,000 TSH)**	10 (45%)

[a] Includes stated willingness to pay preferences for 22 out of 23 participants. Excludes one participant with no stated preference of willingness or payment amount

**Table 3:** Selected quotations of cost advantages from male participants by theme.

Cost Advantages		
Theme	Selected Quotation	Label
**Affordability relative to private clinics**	“…The advantage is there is no need to go to the hospital to take the test. And also, you save money because unlike government hospitals, in private hospitals you must pay to be tested.”	Age 22, PT
	“There are places [where] you pay 3,000 [Tanzanian shillings] and there are places [where] it is just free. In private hospitals, you pay 3,000. And [a] few days back, you can talk to the concerned person and pay 1,000 shillings instead. Yeah, it is private. For example, these caravans on the roads test for free.”	Age 23, NT
**Increased time for earning and other activities**	“So for twenty minutes… And then I have other hours to do different activities around home without wasting more time to go to the clinic and do the test. So, it becomes really easy.”	Age 26, NT
	“The costs to move from here to another place. It becomes a bit easy…Not only [eating] chips but you can take an empty tin and urinate in the vehicle. Eh! You don’t get time! So, if that instrument is available, it becomes a bit simple.”	Age 28, NT
	“Ah, I don’t have much to say because as I said you can go to the hospital and meet a queue and still he does not trust himself. But, if he gets an instrument like this, and uses it alone at home, he can get rid of the hospital queues.”	Age 29, PT
**Avoidance of spending money to test in facilities**	“It is different from making the process of going to the clinic. Therefore, the number of people going to the clinic will decrease. And your personal daily budget, which you reserve, you will be able to buy the instrument because when you go to test at the clinic you incur costs like bus fare, eating and staying in queues. So those costs are reduced a bit.”	Age 28, NT
	“It will help to save time and money because when you come from home and go to test, first of all, there is a queue there. So, you will have to wait. Secondly, you will give money for fare.”	Age 21, NT
	“It will help you to reduce the costs and time which you will spend there as you will just easily go to buy it from the shop and test yourself. Then you will have discovered yourself, and it is not a problem.”	Age 28, NT
**Omission of fees for follow-up visits**	“Yeah, it helps. You test yourself. And once you are done, you throw it in the dustbin. In that way there is no follow up visits.”	Age 26, NT
	“Yeah, like none sees me while I test. And once I am done, I throw it to the dustbin. And in the streets I return – forgetting everything.”	Age 26, NT
	“Even if I see myself that I have done this here. And if I see two lines have appeared, I will be ready to move from this place [home] to the responsible place [clinic] to verify my results.”	Age 28, NT
**Imbalance of benefits of free facility services with costs to access them**	“Yes, it is free completely as I have tested all those times, but I have never been charged except my own fare money…If it is going to Magomeni [hospital], it costs 400 [Tanzanian shillings] going and 400 returning.”	Age 36, PT
	“Like now they have established a lot of testing centers for us and are free. For instance, in short at Mabibo, Mburahati and Mwananyamala, the testing is free. So they help us, unlike in the past where it was until you pay. But nowadays the services have been brought closer. Therefore, it has become a bit better as they have improved certain things.”	Age 26, NT
	“First of all from how I see and from how our country is, it is still not yet. But in developed countries, these things [i.e., HIVST kits] are there. So if the services are close to people, it will be of useful, unlike going to get the services from far places.”	Age 28, NT

NT: Never Tested; PT: Has Been Prior Tested

**Table 4:** Selected quotations of cost disadvantages from male participants by theme.

Cost Disadvantages		
Theme	Selected Quotation	Label
**Preference for free provider-performed tests**	“I don’t see the costs [of HIV testing] but perhaps when the syringe is used on you that are what I see is the costs…It is just a little pain. [But], the cost of money, I don’t know about the side of the NGOs or the government but for example, a few days ago we tested here freely and there was a day we wanted to test but they told me that I should test another day, but I was not told to give money. In the far past, I heard that people were being tested by paying money. But, I have never tested by paying money.”	Age 30, PT
	“You can buy it if you have got that money but from how the economy is, that is, from how the environment is you can go to the clinic.”	Age 28, NT
	“I can just go to the hospital in order to make it easy. 20,000 [for HIVST] is a high price. [Interviewer asks: “So is it better to go to test for free at the hospital?] Yes, because all tests are the same, you want to know about your health so it means testing yourself and going to the hospital are the same.”	Age 23, NT
**Prohibitive and expensive kit costs**	“So if the test is 15,000 [Tanzanian shillings] which is expensive to him and he may find it difficult to pay 15,000. So a person like that how do we help him? The price has to go down a bit so that we all benefit.”	Age 26, NT
	“I may not manage to buy it because that is also a lot of money and testing for HIV is a free will issue. Therefore, at times someone may ignore it perhaps because he has seen it is a lot of money.”	Age 21, NT
	“It will be better because if it is in the low price even the number of people will increase.”	Age 23, NT
	“That’s why I said that if it is sold at a lower price like from 15,000 to 20,000 people will be able to buy it. But, if it will be sold at a higher price like at 30,000 to 40,000, there are others who will fail to buy it – as someone may have the ability to buy it, but says why should I buy it? But at a lower price, a person is able to buy it.”	Age 28, NT
**Required prior savings and value**	“I can tell you that I can buy it at 15,000 [Tanzanian shillings], but I have not prepared myself for that money today.”	Age 31, NT
	“…Your personal daily budget which you reserve, you will be able to buy the instrument…”	Age 28, NT
	“The help I will need [from] my fellow brother unless it costs less, because the way I have checked it there I need it.”	Age 28, NT
**Concerns regarding psychological costs of inaccurate results**	“You understand, one is told [he] is HIV positive, and then the same person goes somewhere else and be told that he/she is negative. Now what if I self-test alone and [it] shows that [I] am not positive while I actually am?”	Age 26, NT
	“Eh, perhaps there may be a certain mistake which I have made or there may be something which I have done wrong? Because I may do it wrong and it shows me that I am HIV negative while I am HIV positive. So, [I] don’t trust myself. “	Age 28, PT
**Concerns of death-related and unwise expenditures**	“It is similar with buying death. It is like some going to buy a poison for suicide! So, I do not know whether the poison is right or wrong. The point of buying it is, like I said, buying my own death. I mean just do not sell it. As none will buy it. If it is sold, it will be hard for someone to decide to go buy it. Trust me. You will go buy your death, I tell you.”	Age 26, NT
	“So you tell me this thing is of good quality and results, but even in hospitals their machines are not working effectively. You know those things are just like any other thing. So, they may fail to work. It is just something that is manufactured, and it may or may not work properly.”	Age 26, NT
	“Many of them can accept, but others may refuse to use it and opt to go to see the doctor. Because, if you understand it well, you can use it. But if you don’t understand it well, it’s better to go to the doctor.”	Age 29, PT

NT: Never Tested; PT: Has Been Prior Tested
